# Impacts of different culture times on pregnancy outcomes after thawing of cleavage stage embryos

**DOI:** 10.1186/s12884-023-06139-7

**Published:** 2023-11-29

**Authors:** Jieyou Wang, Linna Ma, Jiaoqi Mei, Linjiang Li, Wen Xu, Weimin Jiang, Yueyan Wei, Yu Xu, Shaoqing Sun, Yanlin Ma, Qi Li

**Affiliations:** 1 Hainan Modern Women and Children’s Hospital, 18 Qiongzhou Road, Haikou, 570100 China; 2 Hainan Provincial Key Laboratory for Human Reproductive Medicine and Genetic Research, Hainan Clinical Research Center for Thalassemia, Haikou Key Laboratory for Preservation of Human Genetic Resource, Reproductive Medical Center, National Center for International Research “China-Myanmar Joint Research Center for Prevention and Treatment of Regional Major Disease”, The First Affiliated Hospital of Hainan Medical University, Hainan Medical University, Haikou, 570102 China; 3 Key Laboratory of Tropical Translational Medicine of Ministry of Education, Hainan Medical University, 3 Xueyuan Road, Haikou, 571199 China

**Keywords:** Culture times, Cleavage embryos, Frozen-thawed embryo transfer, Pregnancy outcomes

## Abstract

**Objective:**

This study assessed the impacts of in vitro culture times of cleavage embryos on clinical pregnancy outcomes.

**Methods:**

This retrospective cohort study was performed at the Reproductive Medicine Department of Hainan Modern Women and Children’s Hospital in China between January 2018 and December 2022. Patients who first underwent frozen embryo transfer with in vitro fertilization/intracytoplasmic sperm injection (IVF/ICSI) cycles on day 3 were included. According to the time of embryo culture after thawing, the embryos were divided into long-term culture group(18-20 h) and short-term culture group (2-4 h). The clinical pregnancy rate was regarded as he primary outcome. To minimize confounding factors and reduce selection bias, the propensity score matching was used to balance the effects of known confounding factors and to reduce selection bias. Stratified analyses and multiple logistic regression analyses were used to evaluate the risk factors affecting the clinical pregnancy outcomes after matching.

**Results:**

General characteristics between two groups were comparable after matching. In the long-term culture group, 266/381 (69.81%) embryos had more than 10 blastomeres, and 75/381 (19.68%) reached the morula stage. After overnight culture, the implantation rate (27.97% vs. 14.28%, *P =* 0.018) and clinical pregnancy rate (38.46% vs. 22.5%, *P =* 0.05) were increased in the group with proliferating blastomeres. The long-term culture group trended to have a higher clinical pregnancy rate compared with the short-term culture group (35.74% vs. 29.79%). No statistical differences in clinical pregnancy outcomes between the two groups were observed after matching, including the rates of implantation (25.46% vs23.98%), miscarriages (25% vs. 22.85%), ongoing pregnancy rate (76.2% vs. 77.15%) and live birth rate (26.8% vs. 22.98%). Stratified analyses were performed according to the age of the patients. After matching, there were no significant differences in the clinical pregnancy, implantation and miscarriage rates between the two groups for patients > 35 or ≤ 35 years of age. Subgroup analyses were performed according to the quality of the transferred embryos. There were no significant differences in the clinical outcomes, between two groups after embryos transferred with the same quality. Multivariate Logistic regression analysis was used to evaluate the influencing factors of clinical pregnancy outcomes after matching. Culture time was not found to be an independent predictor for clinical pregnancy [OR 0.742, 95%CI 0.487 ~ 1.13; P = 0.165]. The age of oocyte retrieval [OR 0.906, 95%CI 0.865 ~ 0.949; P <0.001] and the number of high-quality embryos transferred [OR 1.787, 95%CI 1.256 ~ 2.543; P = 0.001] were independent factors affecting clinical pregnancy outcomes.

**Conclusions:**

In vitro 18–20 h culture of embryos with either good-or non-good-quality will not adversely affect the clinical pregnancy.

**Supplementary Information:**

The online version contains supplementary material available at 10.1186/s12884-023-06139-7.

## Introduction

With the continuous development of assisted reproductive technology (ART), the proportion of frozen-thawed embryo transfer (FET) has been increasing yearly. According to the statistics reported by The the European Society of Human Reproduction and Embryology (ESHRE), FET accounted for 44% of all embryo transfers in 2016 [1]. Emerging evidence shows that the slow-freezing approach is gradually replaced by vitrification, the latter of which has the advantages of ultra-rapid cooling-warming rates, less labor cost, higher embryo cryo-survival rates and better clinical outcomes compared to the slow-freezing approach [2, 3]. The vitrification protocol has been continuously developed in recent years. Chen et al. showed that FET was associated with a higher live birth rate than fresh embryo transfer in infertile women with polycystic ovary syndrome (PCOS) [4]. The possible reason is that FET cycles allows the ovary to recover from ovarian stimulation, avoiding the adverse effects of supra-physiologic hormonal levels on endometrial receptivity during the controlled ovarian stimulation. On the other hand, exposed endometrial lining shedding allows for better synchronization between embryo and endometrium [5–7]. In addition, embryo cryopreservation prevents the occurrence of ovarian hyperstimulation syndrome and preserves embryos that cannot be transferred due to elevated progesterone in the late follicular phase [8, 9]. A retrospective study showed that compared with cleavage-stage FET, blastocyst FET had significantly increased live birth rates and decreased miscarriage rates [10]. However, many patients still cryopreserve cleavage stage embryos for subsequent frozen-thawed embryo transfer in clinical work. In addition, studies have shown a wide range of blastocyst formation rates, ranging from 4.9 to 66.0% [11]. The cycle cancellation rate was increased during blastocyst cycles as there were no available embryos [12]. Besides, prolonged culture increases the cost of patients [13].

Embryologists have made attempts to improve the embryo implantation potential such as selecting embryos with a high morphological score for thawing [14], improving in vitro embryo culture environments [15], and exploring suitable time for culture in vitro after thawing [16, 17]. At present, few studies were performed to study the in vitro culture time of cleavage embryos after thawing, and the findings are controversial. Jin et al. and Zhao et al. reported that extended culture of cleavage embryos provided a flexible warming/thawing procedure, reduced the spontaneous abortion rate and increased the live birth rate [16, 18]. However, Agha-Rahimi et al. found that there was no significant difference in pregnancy outcome between the long-term culture group (i.e. embryos were warmed and cultured overnight) and the short-term culture group (i.e. embryos were warmed on the day of embryo transfer) [19].

This study investigated the effect of in vitro culture time of cleavage embryos on the clinical pregnancy outcomes, with an aim to provide more direct evidence to guide clinical practice.

## Materials and methods

### Patients and design

This retrospective cohort study on the transfer of vitrified/warmed cleavage stage embryos was performed at the Reproductive Medicine Department of Hainan Modern Women and Children’s Hospital in China between January 2018 and December 2022. Patients who underwent frozen embryo transfer with IVF/ICSI cycles on day 3 were included. Not the first FET cycle, severe uterine anomalies, untreated hydrosalpinx, recurrent miscarriage, diabetes, abnormal thyroid function et were excluded from this study. As a result, a total of 777 cycles with 1356 cleavage stage embryos that were thawed and cultured were enrolled in the final analysis. According to the time of embryo culture after thawing, the embryos were divided into a long-term culture group **(18-20 h)** and a short-term culture group (2-4 h). Ethical approval for the study was obtained from Hainan Modern Women and Children’s hospital.

### Ovarian stimulation and oocyte retrieval

Controlled ovarian hyperstimulation was carried out with a long GnRH agonist or antagonist protocol other protocols, including mild stimulation and luteal phase stimulation protocols according to the patient age and ovarian reserve [20]. When one dominant follicle was ≥ 20 mm or two dominant follicles reached an average diameter of ≥ 18 mm, HCG (5000-10,000 IU) was administered. Oocytes were then collected after 36–38 h. Oocytes were fertilized by standard IVF/ ICSI procedures. Fertilization assessment was conducted 16–20 h after insemination.

### Embryo culture, vitrification and warming, embryo score

Fertilized oocytes were individually cultured in G-1 Plus medium drop (Vitrolife, Sweden) for 3 days. On Day 3, embryo morphology was evaluated according to the number of cells, regularity of blastomere and degree of fragmentation [21]. The assessment of the embryo was conducted by two proficient embryologists, and the contentious aspect was determined by the more skilled physician. Grade levels were defined as follows: Grade I: embryo had even blastomeres, integrated zona pellucida, fragments＜5%, and without vacuoles and multi-nucleation; Grade II: embryo had even or uneven blastomeres, integrated zona pellucida, fragments between 5 and 20%, and without vacuoles and multi-nucleation; Grade III: embryos had uneven blastomere, few vacuoles, the abnormal zona pellucida, fragmentation covering 20 and 50% of the embryo surface; and Grade IV: embryos contained few blastomeres of any size and severe fragmentation covering. Grade I or II embryos were considered as good quality embryos. Embryos were cryopreserved by vitrification on the third day after oocyte retrieval. The cleavage stage embryo vitrification and warming protocols were performed following the vitrification procedure according to the instructions of Cryotop Safety Kit (Kitazato, Japan). Briefly, each embryo was placed in the equilibration solution for 10–12 min. The equilibration solution contained 7.5% (v /v) ethylene glycol and 7.5% (v/v) dimethyl sulfoxide. Embryos were then transferred to the vitrification solution (15% (v/v) ethylene glycol and 15% (v/v) dimethyl sulfoxide for about 45-60s. Finally, embryos were placed on the Cryotop and stored in liquid nitrogen. During warming, the Cryotop was quickly removed from liquid nitrogen. Embryos were immersed into thawing solution (1.0 M sucrose) for one minute, then gently transferred to diluent solution (0.5 M sucrose) at room temperature for 3 min. Thereafter, embryos were washed twice in washing solution at room temperature for 5 min each time, then placed in culture dishes containing G-2 Plus culture medium and incubated in an incubator with 37 °C, 6% CO_2_,5% O_2_ and 89% N_2_ for complete recovery.

### Endometrium preparation and transfer

Endometrium preparation protocols included both natural and artificial cycles. For patients with a regular menstrual cycle and ovulation, the natural cycle was used. During the 10 days of menstrual cycle, and transvaginal ultrasound was preformed to monitor follicular development and endometrium. When the diameter of a dominant follicle was ≥ 14 mm, serum E_2_, LH and progesterone levels were measured to determine ovulation. After ovulation, progesterone injection was administered daily. For patients with an irregular menstrual cycle, the artificial cycle was applied. Estradiol valerate was administered from the second to the third day of menstruation, and the dose was adjusted according to the endometrial thickness. When the endometrial thickness was ≥ 7 mm orestradiol valerate was taken for more than 12 days, patients were treated with progesterone for endometrial transformation. All patients underwent embryo transfer under abdominal ultrasound guidance on the third day of progesterone administration. All patients received luteal phase support after frozen embryo transfer.

### Luteal phase support

The prescribed luteal support regimen is outlined as follows: The administration of luteal support was initiated within three days following natural ovulation or ovulation induced by human chorionic gonadotropin (hCG) in patients who underwent frozen-thawed embryo transfer in either natural cycles or ovulation induction cycles. When considering factors such as drug safety, drug cost, patient choice satisfaction, and patient compliance, it is important to carefully evaluate and pick the most suitable method of administration for supplementing progesterone. One option for oral progesterone is to take oral dydrogesterone at a dosage of 30 mg per day. Another option is to take oral micronized progesterone capsules at a dosage of 200–300 mg per day, either once or twice daily. It is important to note that a single dose should not exceed 200 mg. Progesterone for vaginal application can be administered through two methods: progesterone vaginal sustained-release gel at a dosage of 90 mg per day, and progesterone micronized capsules at a dosage of 600 mg per day, divided into three vaginal administrations. It is important to note that a single dose should not exceed 200 mg. The administration of progesterone via intramuscular injection at a dosage of 20 mg per day. Patients undergoing hormone replacement therapy with frozen embryo transfer (HRT-FET) initiated luteal support concurrent with endometrial transformation. Progesterone supplementation was commenced by the third day, 3 to 5 days prior to embryo transplantation. The administration and dosage of different progesterone preparations were as follows.

### Clinical outcomes

The primary outcome was defined as the clinical pregnancy (the presence of an intrauterine gestational sac on the 30th day). The secondary outcomes included the implantation rate (the number of gestational sacs after embryo transfer divided by the number of transferred embryos), and the miscarriage rate (the number of cycles of abortion divided by the total number of pregnancy cycles) Ongoing pregnancy rate (the number of cycles of pregnancy lasting 20 weeks or more/the total number of pregnancy cycles), live birth rate(number of live births/the number of transferred cycles).

### Statistical analysis

Data were analyzed using SPSS version 22.0 and R 3.3.3 software. Measurement data conforming to normal distribution were presented as mean ± standard deviation (SD) and compared with the student’s t-test between two groups. Non-normally distributed measures were expressed as median and interquartile spacing [M(Q1, Q3)], and comparisons between groups were made by rank sum test. Enumeration data were shown by the rate (%) and compared with the χ2 test or Fisher’s exact test, whichever was applicable. No-release propensity score matching was performed according to 1:1, and the caliper value was set to 0.05. The inter-group balance was evaluated by standard differences, and if the standard difference < 10%, it is considered that the balance between the variables was considered better. Covariates such as BMI, FSH, AFC, AMH, endometrial preparation protocol, endometrial thickness, number of transplanted embryos, and quality of transplanted embryos were used for propensity matching. Multivariate logistic regression analysis was used to evaluate the influencing factors of clinical pregnancy after matching. A *p* value less than 0.05 was considered statistically significant.

## Results

**Fig. 1:**
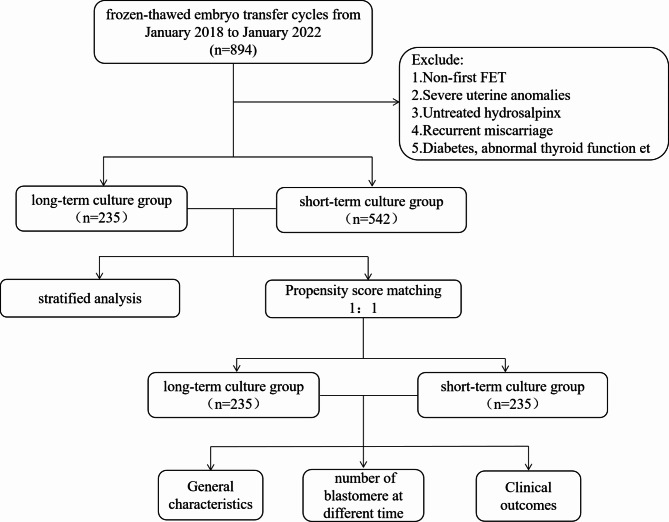
Flowchart of the study

**Fig. 2:**
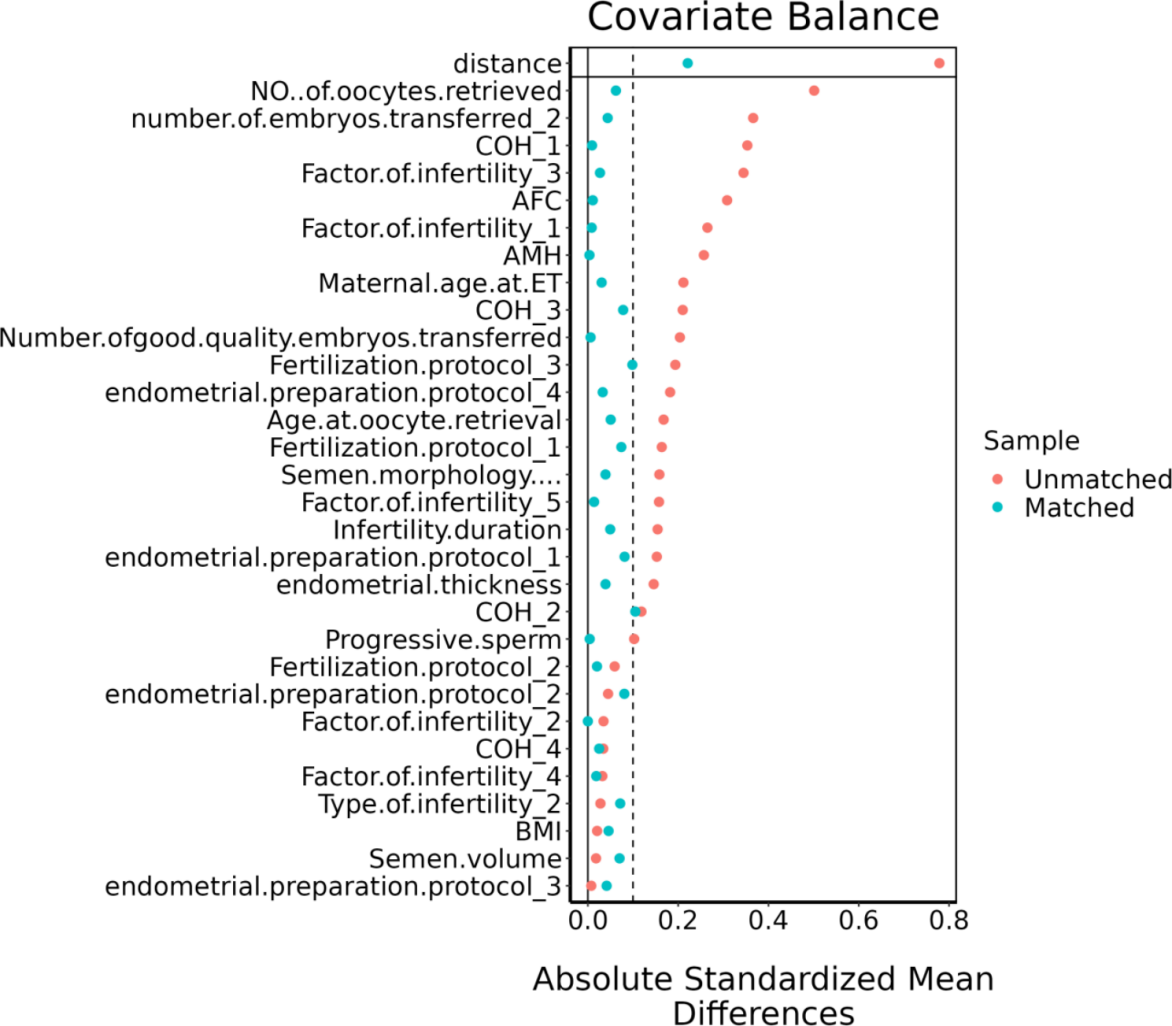
Scatterplot of standardized differences for each covariate

A total of 777 first FET cycles were analyzed in this study, all of which were at the cleavage stage. Cycles were divided into the long-term culture group (n = 235) and the short-term culture group (n = 542) based on the time of in vitro culture (Fig. 1). General characteristics of embryos in these two groups are shown in Table 1. The median ages of oocyte retrieval in the two groups were 36.09 ± 5.22 and 35.21 ± 5.32 years, respectively. There were significant differences between the two groups in terms of the age of oocyte retrieval or ET, infertility factors, AMH, number of embryos transferred(P＜0.05).Propensity score matching was subsequently performed to minimize the imbalance of baseline characteristics.235 patients remained in both groups after matching. The basal characteristics after matching are also presented in Table 1. None of them demonstrated a significant difference between groups. (all *P* values > 0.05) (Table 1), and the covariates were well balanced between the groups (Fig. 2).

**Table 1:** General characteristics before and after matching

Variables	Before Matching				After Matching			
	Long-term culture group (n = 235)	Short-term culture group (n = 542)	SMD^∆^	*P value*	Long-term culture group (n = 235)	Short-term culture group (n = 235)	SMD^∆^	*P value*
Age at oocyte retrieval	36.09 ± 5.22	35.21 ± 5.32	0.168	***0.03***	36.09 ± 5.22	36.13 ± 5.31	-0.008	*0.93*
Maternal age at ET	36.66 ± 5.15	35.56 ± 5.33	0.212	***0.00***	36.66 ± 5.15	36.60 ± 5.30	0.012	*0.90*
Infertility duration	5.86 ± 4.04	5.24 ± 3.92	0.155	***0.04***	5.86 ± 4.04	5.63 ± 4.00	0.058	*0.52*
Type of infertility, n (%)				*0.72*				*0.19*
Primary	81 (34.5)	194 (35.8)	-0.028		81 (34.5)	68 (28.9)	0.116	
Secondary	154 (65.5)	348 (64.2)	0.028		154 (65.5)	167 (71.1)	-0.116	
Factor of infertility				***0.00***				*0.73*
Tubal factor	101 (43.0)	304 (56.1)	-0.265		101 (43.0)	111 (47.2)	-0.086	
PCOS	15 (6.4)	30 (5.5)	0.035		15 (6.4)	12 (5.1)	0.052	
DOR	81 (34.5)	98 (18.1)	0.345		81 (34.5)	71 (30.2)	0.090	
Endometriosis	13 (5.5)	26 (4.8)	0.032		13 (5.5)	17 (7.2)	-0.074	
Other	25 (10.6)	84 (15.5)	-0.158		25 (10.6)	24 (10.2)	0.014	
BMI (kg/m^2^)	22.62 ± 3.02	22.56 ± 2.94	0.020	*0.78*	22.62 ± 3.02	22.48 ± 2.77	0.048	*0.58*
AFC (n)	9.97 ± 7.02	12.14 ± 6.70	-0.308	***0.00***	9.97 ± 7.02	9.88 ± 5.81	0.014	*0.86*
AMH (ng/ml)	2.10 ± 2.31	2.70 ± 2.82	-0.257	***0.00***	2.10 ± 2.31	2.08 ± 2.75	0.010	*0.92*
COH Protocol				***0.00***				*0.93*
Long GnRH agonist protocol	70 (29.8)	249 (45.9)	-0.353		70 (29.8)	69 (29.4)	0.009	
Antagonist protocols	64 (27.2)	119 (22.0)	0.119		64 (27.2)	60 (25.5)	0.038	
PPOS	94 (40.0)	161 (29.7)	0.210		94 (40.0)	97 (41.3)	-0.026	
Other	7 (3.0)	13 (2.4)	0.034		7 (3.0)	9 (3.8)	-0.050	
NO. of oocytes retrieved	7.69 ± 6.09	10.74 ± 7.74	-0.501	***0.00***	7.69 ± 6.09	7.64 ± 5.70	0.008	*0.93*
Semen volume (mL)	3.14 ± 1.34	3.16 ± 1.84	-0.020	*0.84*	3.14 ± 1.34	3.22 ± 1.84	-0.063	*0.56*
Progressive sperm	36.15 ± 18.69	34.21 ± 18.07	-0.107	*0.18*	34.21 ± 18.07	31.80 ± 17.90	0.133	*0.14*
Semen morphology (%)	3.59 ± 2.02	3.90 ± 2.34	-0.152	*0.05*	3.59 ± 2.02	3.25 ± 1.79	0.176	*0.05*
Fertilization protocol, n (%)				***0.00***				*0.16*
IVF	164 (69.8)	419 (77.3)	-0.164		164 (69.8)	175 (74.5)	-0.102	
ICSI	54 (23.0)	111 (20.5)	0.059		54 (23.0)	111 (20.5)	0.020	
IVF + ICSI	17 (7.2)	12 (2.2)	0.194		17 (7.2)	8 (3.4)	0.148	

PCOS: polycystic ovarian syndrome; DOR: Diminished ovarian reserve; BMI: body mass index; AFC: antral follicle count; AMH: Anti-mullerian hormone; COH: controlled ovarian stimulation; PPOS: progestin primed ovarian stimulation; IVF: in vitro fertilization; ICSI: intracytoplasmic sperm injection; SMD: Standardized Mean Difference

**Fig. 3:**
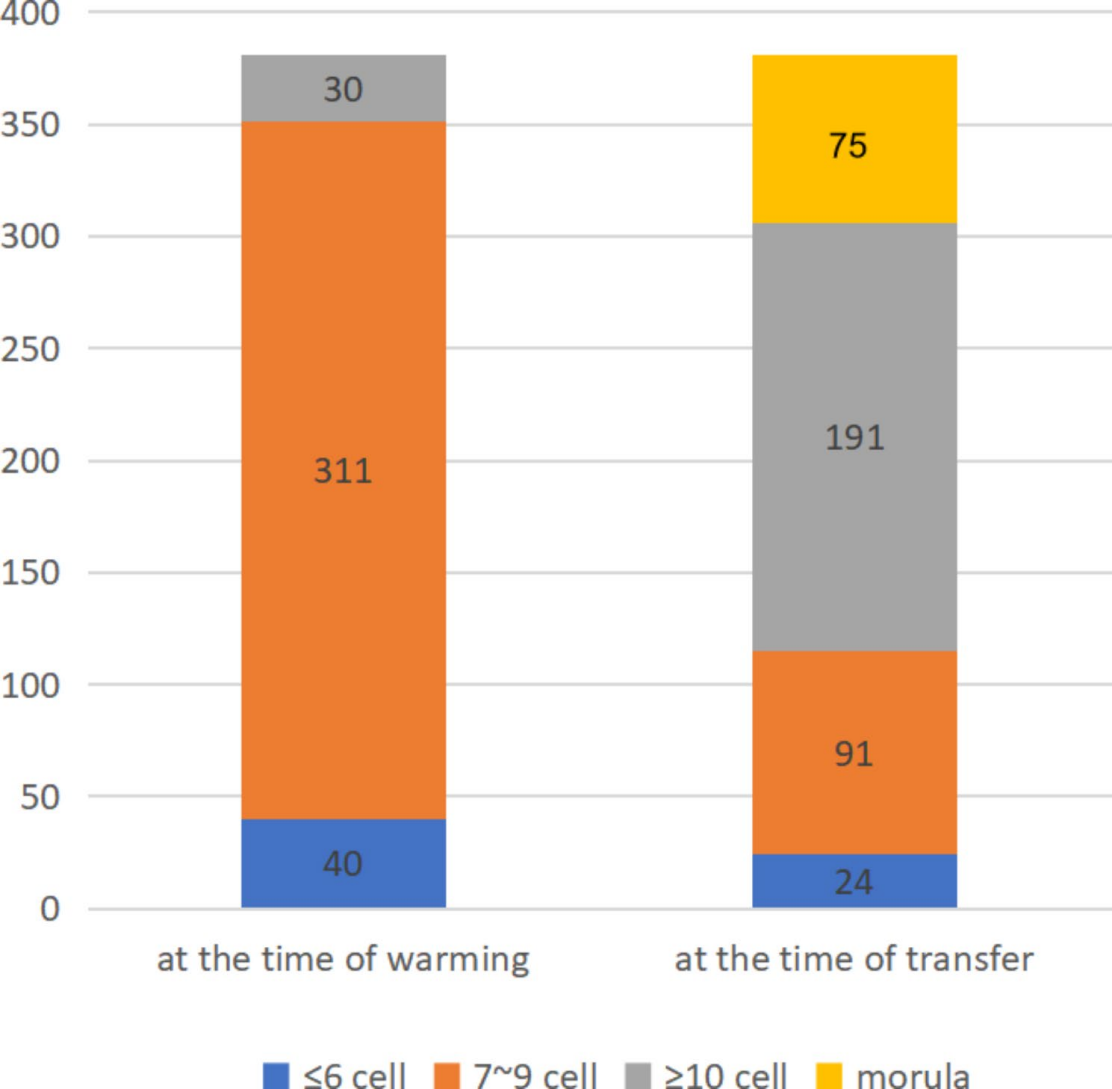
Changes in the number of blastomere at different time points in the long-term culture group

The majority of cleavage stage embryos were observed to proliferate in the long-term culture group. In total, 266/381 (69.81%) embryos developed more than 10 blastomeres, and 75/381(19.68%) reached the morula stage (Fig. 3). Compared with the non-proliferation group, the implantation rate **(**27.97% vs14.28%**)** and clinical pregnancy rate (38.46% vs22.5%%) of the proliferation group increased (Table 2).

**Table 2:** The effect of cell proliferation on clinical outcome after overnight culture

	Implantation rate	*P* value	Clinical pregnancy rate	*P* value	Miscarriage rate	*P* value
proliferation group	87/311(27.97%)	***0.01***	75/195(38.46%)	***0.05***	19/75(25.33%)	*0.83*
unproliferated group	10/70(14.28%)		9/40(22.5%)		2/9(22.22%)	

Table 3 presents treatment characteristics of embryos in two groups before and after matching. The endometrial preparation protocol, the number of embryos transferred and the number of top-quality embryos transferred between the two groups were comparable after matching. Although the long-term culture group had a tendency to have a higher clinical pregnancy rate, the difference was not statistically different between the two groups (**35.74%** vs. **29.79**%). There were no significant differences in the implantation rate (25.46% vs23.98%), miscarriage rate (25% vs. 22.85%), ongoing pregnancy rate (76.2% vs. 77.15%) and live birth rate (26.8% vs. 22.98%) between these two groups.

Stratified analyses were then performed according to the age of the patients. After propensity matching scores, there was no overall difference in the general characteristics between subgroups (data are shown in supplementary I). As expected, the implantation rate and clinical pregnancy rate of patients ≤ 35 years old in the two groups were higher. After matching, there were no significant differences in implantation, clinical pregnancy, or miscarriage rates between the two groups for patients > 35 or ≤ 35 years of age (Tables 4 and 5).

**Table 3:** Clinical outcomes of FETs in two groups before and after matching

Variables	Before Matching				After Matching			
	Long-term culture group (n = 235)	Short-term culture group (n = 542)	SMD^∆^	*P* value	Long-term culture group (n = 235)	Short-term culture group (n = 235)	SMD^∆^	*P* value
Endometrial preparation protocol (%)				*0.05*				*0.70*
Natural cycles	26 (11.1)	34 (6.3)	0.153		26 (11.1)	20 (8.5)	0.081	
HRT cycles	155 (66.0)	369 (68.1)	-0.045		155 (66.0)	155 (66.0)	0.000	
GnRH agonist-HRT	50 (21.3)	117 (21.6)	-0.008		50 (21.3)	57 (24.3)	-0.073	
Other	4 (1.7)	22 (4.1)	-0.182		4 (1.7)	3 (1.3)	0.033	
Endometrium thickness(mm)	9.47 ± 1.89	9.20 ± 1.47	0.146	*0.04*	9.47 ± 1.89	9.48 ± 1.56	-0.001	*0.98*
Number of embryos transferred				*0.00*				*0.29*
1(%)	89 (37.9)	109 (20.1)	0.366		89 (37.9)	78 (33.2)	0.096	
2(%)	146 (62.1)	433 (79.9)	-0.366		146 (62.1)	157 (66.8)	0.096	
Number of good-quality embryos transferred	1.26 ± 0.70	1.41 ± 0.72	-0.204	*0.01*	1.26 ± 0.70	1.28 ± 0.71	-0.024	*0.79*
Implantation rate (%)	97/381(25.46)	225/975(23.08)		*0.35*	97/381(25.46)	94/392(23.98)		*0.63*
Clinical pregnancy rate (%)	84/235(35.74)	175/542(32.29)		*0.34*	84/235(**35.74**)	70/235(**29.79**)		*0.16*
Miscarriage rate (%)	21/84(25)	43/175(24.57)		*0.95*	21/84(25)	16/70(22.85)		*0.75*
Ongoing pregnancy rate(%)	64/235(27.23)	135/542(24.91)		*0.50*	64/235(27.23)	54/235(22.98)		*0.29*
Live birth rate(%)	63/235(26.80)	132/542(24.35)		*0.47*	63/235(26.80)	54/235(22.98)		*0.34*

**Table 4:** Clinical outcomes of FET in patients ≤ 35 years of age after matching

	Variables	Before Matching				After Matching			
		Long-term culture group (n = 102)	Short-term culture group (n = 255)	SMD^∆^	*P* value	Long-term culture group (n = 102)	Short-term culture group (n = 102)	SMD^∆^	*P* value
≤ 35 year	Implantation rate (%)	63/173(36.42)	139/470(29.57)		***0.10***	63/173(36.42)	50/176(28.41)		*0.11*
	Clinical pregnancy rate (%)	52/102(50.98)	106/255(41.57)		0.10	52/102**(50.98)**	40/102(**39.22**)		*0.09*
	Miscarriage rate (%)	11/52(21.15)	19/106(17.92)		*0.62*	11/52(21.15)	11/40(27.5)		*0.47*

**Table 5:** Clinical outcomes of FET in patients > 35 years of age after matching

	Variables	Before Matching				After Matching		
		Long-term culture group (n = 133)	Short-term culture group (n = 287)		*P* value	Long-term culture group (n = 133)	Short-term culture group (n = 133)	*P* value
＞35 year	Implantation rate (%)	34/208(16.35)	86/505(17.03)		*0.91*	34/208(16.35)	37/229(16.16)	*0.95*
	Clinical pregnancy rate (%)	32/133(24.06)	69/287(24.04)		*0.99*	32(24.06)	29(21.80)	*0.66*
	Miscarriage rate (%)	10/32(31.25)	24/69(34.78)		*0.72*	10/32(31.25)	10/29(34.48)	*0.58*

Subgroup analyses were then performed according to the quality of embryos. The inter-group comparison showed the implantation rate (30.32%, 31%, respectively) and clinical pregnancy rate (52.13%, 42%, respectively) were higher in two good quality embryos of transferred group. There were no significant differences in the clinical pregnancy rate, implantation rate and miscarriage rate between the two groups after embryos with the same quality were transferred (Table 6).

**Table 6:** Pregnancy outcomes of FETs in Subgroup analyses

		Implantation rate			Clinical pregnancy rate			Miscarriage rate		
Number of embryos transferred	Number of good quality embryos transferred	long-term culture group	short-term culture group	*P* value	long-term culture group	short-term culture group	*P* value	long-term culture group	short-term culture group	*P* value
2	0	11/46(23.91%)	6/44(13.63%)	*0.21*	9/23(39.13%)	5/22(22.72%)	*0.23*	2/9(22.22%)	3/5(60%)	*0.40*
2	1	3/58(5.17%)	10/70(14.28%)	*0.08*	3/29(10.34%)	8/35(22.85%)	*0.32*	0/9	1/8(12.5%)	*0.47*
2	2	57/188(30.32%)	62/200(31%)	*0.88*	49/94(52.13%)	42/100(42%)	*0.15*	13/49(26.53%)	9/42(21.42%)	*0.57*
1	0	0/10	1/13(7.69%)	*-*	0/10	1/13(7.69%)	*-*	0/0	0/1	*-*
1	1	24/79(30.38%)	15/65(23.07%)	*0.32*	23/79(29.11%)	14/65(21.54%)	*0.30*	6/23(26.08%)	3/14(21.42%)	*0.74*

Multivariate logistic regression analysis was used to evaluate the influencing factors of clinical pregnancy after matching. Variables with known and potential confounders were included in the multifactor binary logistic regression model. Culture time was not found to be an independent predictor for clinical pregnancy [OR 0.742, 95%CI 0.487 ~ 1.13; P = 0.165] after adjusting for age, BMI, endometrial preparation protocol, endometrium thickness, number of embryos transferred and number of good-quality embryos transferred. However, the age of oocyte retrieval [OR 0.906, 95%CI 0.865 ~ 0.949; P < 0.001] and the number of high-quality embryos transferred [OR 1.787, 95%CI 1.256 ~ 2.543; P = 0.001] were independent factors affecting clinical pregnancy (Table 7).

**Table 7:** Multivariate binary logistic regression analysis associations for patients with and without clinical pregnancy

Factor	Regression coefficients(β)	Adjusted OR	Adjusted OR 95% confidence interval	Significance (P)
Age at oocyte retrieval	-0.098	0.90	0.865 ~ 0.949	**0.00**
BMI	0.041	1.04	0.966 ~ 1.123	0.28
Culture time	-0.298	0.74	0.487 ~ 1.13	0.165
NO. of oocytes retrieved	0.049	1.0	1.009 ~ 1.092	**0.01**
Endometrial preparation protocol				0.54
Natural cycles				
HRT cycles	0.178	1.19	0.55 ~ 2.597	0.65
GnRH agonist-HRT	-0.187	0.82	0.35 ~ 1.968	0.67
Other	0.555	1.74	0.285 ~ 10.67	0.54
Endometrial thickness	0.095	1.1	0.973 ~ 1.243	0.12
Number of embryos transferred	0.184	1.20	0.715 ~ 2.018	0.48
Number of good-quality embryos transferred	0.581	1.78	1.256 ~ 2.543	**0.00**

## Discussion

Frozen-thawed (FT) embryo transfer has gained popularity and significance within ART in recent years. The utilization of FET serves as a means to conserve surplus embryos obtained during the processes of IVF/ ICSI, while concurrently mitigating the occurrence of hyperstimulation syndrome [22]. Accumulating evidence suggests that FET can greatly improved birth outcomes. For example, FET leads to fewer postpartum hemorrhages and premature delivery, small for gestational age, lower birth weight, and fewer perinatal fetal deaths compared to fresh embryo transfer [24, 25]. On the other hand, in contrast to fresh blastocyst transfer, IVF/ICSI conceptions with thawed blastocyst transfer present a lower mean uterine artery pulsatility index and greater crown-rump length [26]. Previous studies have indicated that adverse perinatal outcomes in the fresh cycle may be associated with impaired placentation, which may be linked to supraphysiological estradiol levels [27, 28]. However, recent studies have shown that, compared to neonates born after fresh transfers, those newborns after FET had a higher risk of being large for gestational age, having macrosomia, and an increased risk of preeclampsia [29]. Currently, there are no recommendations for implementing a freeze-all strategy but individualization according to patients’ characteristics. It is widely acknowledged that various factors contribute to the clinical pregnancy outcome of frozen embryo transfer (FET). These factors encompass patient age, embryo quality, endometrial receptivity during transplantation, as well as the duration of in vitro culture for frozen-thawed embryos, among others.

In this retrospective study, we aimed to assess the influence of post-thaw culture times of embryos on clinical pregnancy outcomes. We found no statistically significant differences in implantation and miscarriage rates between two groups after 1 : 1 propensity score matching score, however, the long-term culture group had a tendency to have a higher clinical pregnancy rate compared with the short-term culture group. After overnight culture, the implantation rate and clinical pregnancy rate were increased in the group with proliferating blastomeres. This suggests that the proliferated embryos have better clinical outcomes. We conducted stratified analysis based on the age of the patients in the FET cycle, and found that there was no statistical difference in the clinical outcomes between all groups before and after matching. We performed subgroup analysis based on the number of good-quality embryos in the FET cycle, and found that there was no statistical difference in the clinical outcomes between all groups. Our findings suggest that the time of in vitro culture has no effect on clinical outcomes, either good-quality or non-good-quality embryos after thawing. Multivariate logistic regression analysis showed that the time of in vitro culture has no effect on clinical outcomes, which were in line with previously published results by Guo et al. [30]. Guo et al. also showed that the clinical outcomes of two methods (short culture and long culture) were no difference in FET cycles including at least one good-quality embryo. In contrast to our study, Rato et al. showed that implantation rate and live birth rate were higher in the short culture group [31].

Several reasons may underlie the difference between our findings and previous ones. First, Rato et al. showed that implantation rate and live birth rate were higher in short culture group. They used slow freezing to freeze embryos, and the recovery rate of embryos after thawing was reduced. All embryos of patients in our study were processed by vitrification freezing, which avoids the formation of intracellular ice crystals, reduces damage to embryos and improves embryo recovery rate. Second, the prolonged culture time in vitro was inconsistent. Zhao et al. extended the culture time by 7 ~ 8 h. Thirdly, compared with other studies, we use propensity score matching to reduce confounding factors; then subgroup analysis based on embryo quality was more reliable. Finally, the criteria for embryo scoring were different.

Although the conditions of embryo culture in vitro have been greatly improved, embryos are still exposed to stresses including ROS, temperature, and PH, which have a negative impact on embryonic developmental potential [32, 33]. However, more embryonic development information can be obtained during post-thaw overnight culture in FET cycles, such as blastomeres survival, mitosis resumption, number of blastomeres, symmetry and fragmentation [34]. In the present study, most embryos in the long-term culture group were observed to proliferate, and 19.68% were fusion embryos or early morula. The above information can therefore be used to assess embryonic development potential, and the embryos with developmental potential can improve the clinical pregnancy rate and reduce the abortion rate.

Some limitations of this study should be acknowledged. First, this was a retrospective study, which should have some intrinsic disadvantages associated with this study nature. However, we screened two groups by using the propensity score matching method, so that the selected subjects were comparable in clinical characteristics and the results were more reliable. Second, two embryos were transferred in some patients, and it was difficult to distinguish which embryo had successful pregnancy eventually. Our observations from this study need to be corroborated in a randomized controlled trial with a large sample size in the future.

In conclusion, in vitro overnight culture of thawed cleavage embryos will not adversely affect the clinical outcomes of pregnancy. Hence, embryologists can arrange the time flexibly according to the work shifts for FET.

### Electronic supplementary material

Below is the link to the electronic supplementary material.

Supplementary Material 1

## Data Availability

The datasets used and/or analyzed during the current study are available from the corresponding author on reasonable request.
