# Supplementary material for: Impacts of different culture times on pregnancy outcomes after thawing of cleavage stage embryos
Source: BMC Pregnancy Childbirth. 2023 Nov 29;23:824. doi: 10.1186/s12884-023-06139-7 (PMC10685551; doi:10.1186/s12884-023-06139-7)
Supplement: Supplementary file 1 — Supplementary Material 1 [file 12884_2023_6139_MOESM1_ESM.docx]

**Supplementary table 1 General characteristics before and after matching of patients**≤**35 years old**

| Variables | **Before Matching** | | |  | **After Matching** | | |  |
| --- | --- | --- | --- | --- | --- | --- | --- | --- |
| ≤35 years | Long-term culture group  (n=102) | Short-term culture group  (n=255) | SMD^△^ | *P value* | Long-term culture group  (n=102) | Short-term culture group  (n=102) | SMD^△^ | *P value* |
| Age at oocyte retrieval | 31.18±2.55 | 30.52±2.99 | 0.258 | *0.03* | 31.18±2.55 | 31.33±2.62 | -0.062 | *0.66* |
| Maternal age at ET | 31.79±2.50 | 30.84±2.93 | 0.384 | ***0.00*** | 31.79±2.50 | 31.78±2.48 | 0.004 | *0.97* |
| Infertility duration | 4.88±2.73 | 4.35±2.66 | 0.197 | *0.08* | 4.88±2.73 | 4.66±2.65 | 0.083 | *0.55* |
| Type of infertility, n (%) |  |  |  | *0.31* |  |  |  | *0.67* |
| Primary | 56 (54.9) | 125 (49.0) | 0.118 |  | 56 (54.9) | 53 (52.0) | 0.059 |  |
| Secondary | 46 (45.1) | 130 (51.0) | -0.118 |  | 46 (45.1) | 49 (48.0) | -0.059 |  |
| Factor of infertility |  |  |  | *0.00* |  |  |  | *0.89* |
| Tubal factor | 47 (46.1) | 157 (61.6) | -0.311 |  | 47 (46.1) | 52 (51.0) | -0.098 |  |
| PCOS | 12 (11.8) | 24 (9.4) | 0.073 |  | 12 (11.8) | 11 (10.8) | 0.030 |  |
| DOR | 19 (18.6) | 17 (6.7) | 0.307 |  | 19 (18.6) | 14 (13.7) | 0.126 |  |
| Endometriosis | 9 (8.8) | 11 (4.3) | 0.159 |  | 9 (8.8) | 9 (8.8) | 0.000 |  |
| Other | 15 (14.7) | 46 (18.0) | -0.094 |  | 15 (14.7) | 16 (15.7) | -0.028 |  |
| BMI (kg/m^2^) | 22.42±2.94 | 22.11±3.09 | 0.104 | *0.39* | 22.42±2.94 | 22.09±3.04 | 0.113 | *0.43* |
| AFC (n) | 12.82±7.53 | 15.33±6.80 | -0.332 | ***0.00*** | 12.82±7.53 | 12.98±6.44 | -0.021 | *0.87* |
| AMH (ng/ml) | 3.01±2.51 | 3.90±3.26 | -0.357 | ***0.01*** | 3.01±2.51 | 3.31±3.53 | -0.122 | *0.47* |
| COH Protocol |  |  |  | ***0.00*** |  |  |  | *0.64* |
| Long GnRH agonist protocol | 49 (48.0) | 185 (72.5) | -0.491 |  | 49 (48.0) | 54 (52.9) | -0.098 |  |
| Antagonist protocols | 30 (29.4) | 34 (13.3) | 0.353 |  | 30 (29.4) | 22 (21.6) | 0.172 |  |
| PPOS | 22 (21.6) | 34 (13.3) | 0.200 |  | 22 (21.6) | 25 (24.5) | -0.072 |  |
| Other | 1 (1.0) | 2 (0.8) | 0.020 |  | 1 (1.0) | 1 (1.0) | 0.000 |  |
| NO. of oocytes retrieved | 9.96±6.54 | 14.66±8.37 | -0.719 | *0.00* | 9.96±6.54 | 10.53±6.64 | -0.087 |  |
| Semen volume (mL) | 3.19±1.30 | 3.19±1.92 | -0.005 | *0.97* | 3.19±1.30 | 3.16±2.08 | 0.021 | *0.90* |
| Progressive sperm | 32.40±18.26 | 37.38±8.82 | -0.273 | *0.02* | 32.40±18.26 | 35.93±19.20 | -0.193 | *0.18* |
| Semen morphology (%) | 3.61±1.95 | 3.90±2.32 | -0.146 | *0.28* | 3.61±1.95 | 3.68±2.31 | -0.036 | *0.81* |
| Fertilization protocol, n (%) |  |  |  | *0.00* |  |  |  | *0.24* |
| IVF | 66 (64.7) | 196 (76.9) | -0.254 |  | 66 (64.7) | 71 (69.6) | -0.103 |  |
| ICSI | 23 (22.5) | 49 (19.2) | 0.080 |  | 23 (22.5) | 25 (24.5) | -0.047 |  |
| IVF+ICSI | 13 (12.7) | 10 (3.9) | 0.265 |  | 13 (12.7) | 6 (5.9) | 0.206 |  |

**Supplementary table 2 Clinical outcomes of FETs in two groups before and after matching of patients** ≤**35 years old**

| Variables | Before Matching | | |  | After Matching | | |  |
| --- | --- | --- | --- | --- | --- | --- | --- | --- |
| ≤35 years | Long-term culture group  (n=102) | Short-term culture group  (n=255) | SMD^△^ | *P* value | Long-term culture group  (n=102) | Short-term culture group  (n=102) | SMD^△^ | *P* value |
| Endometrial preparation protocol (%) |  |  |  | *0.45* |  |  |  | *0.99* |
| Natural cycles | 8 (7.8) | 17 (6.7) | 0.044 |  | 8 (7.8) | 7 (6.9) | 0.036 |  |
| HRT cycles | 73 (71.6) | 180 (70.6) | 0.022 |  | 73 (71.6) | 74 (72.5) | -0.022 |  |
| GnRH agonist-HRT | 20 (19.6) | 47 (18.4) | 0.030 |  | 20 (19.6) | 20 (19.6) | 0.000 |  |
| Other | 1 (1.0) | 11 (4.3) | -0.338 |  | 1 (1.0) | 1 (1.0) | 0.000 |  |
| Endometrium thickness(mm) | 9.66±2.02 | 9.16±1.41 | 0.246 | *0.02* | 9.66±2.02 | 9.41±1.47 | 0.121 | *0.32* |
| Number of embryos transferred |  |  |  | *0.00* |  |  |  | *0.64* |
| 1(%) | 31 (30.4) | 40 (15.7) | 0.320 |  | 31 (30.4) | 28 (27.5) | 0.064 |  |
| 2(%) | 71 (69.6) | 215 (84.3) | -0.320 |  | 71 (69.6) | 74 (72.5) | -0.064 |  |
| Number of good-quality embryos transferred | 1.35±0.74) | 1.43±0.74 | -0.106 | *0.36* | 1.35±0.74 | 1.36±0.71 | -0.013 | *0.92* |
| Implantation rate (%) | 90/173(52.02) | 200/470(42.55) |  | ***0.00*** | 126/173(72.83) | 128/176(72.72) |  | *0.94* |
| Clinical pregnancy rate (%) | 52/102(50.98) | 106/255(41.57) |  | 0.10 | 52/102(50.98) | 40/102(39.22) |  | *0.09* |
| Miscarriage rate (%) | 11/52(21.15) | 19/106(17.92) |  | *0.62* | 11/52(21.15) | 11/40（27.5） |  | *0.47* |


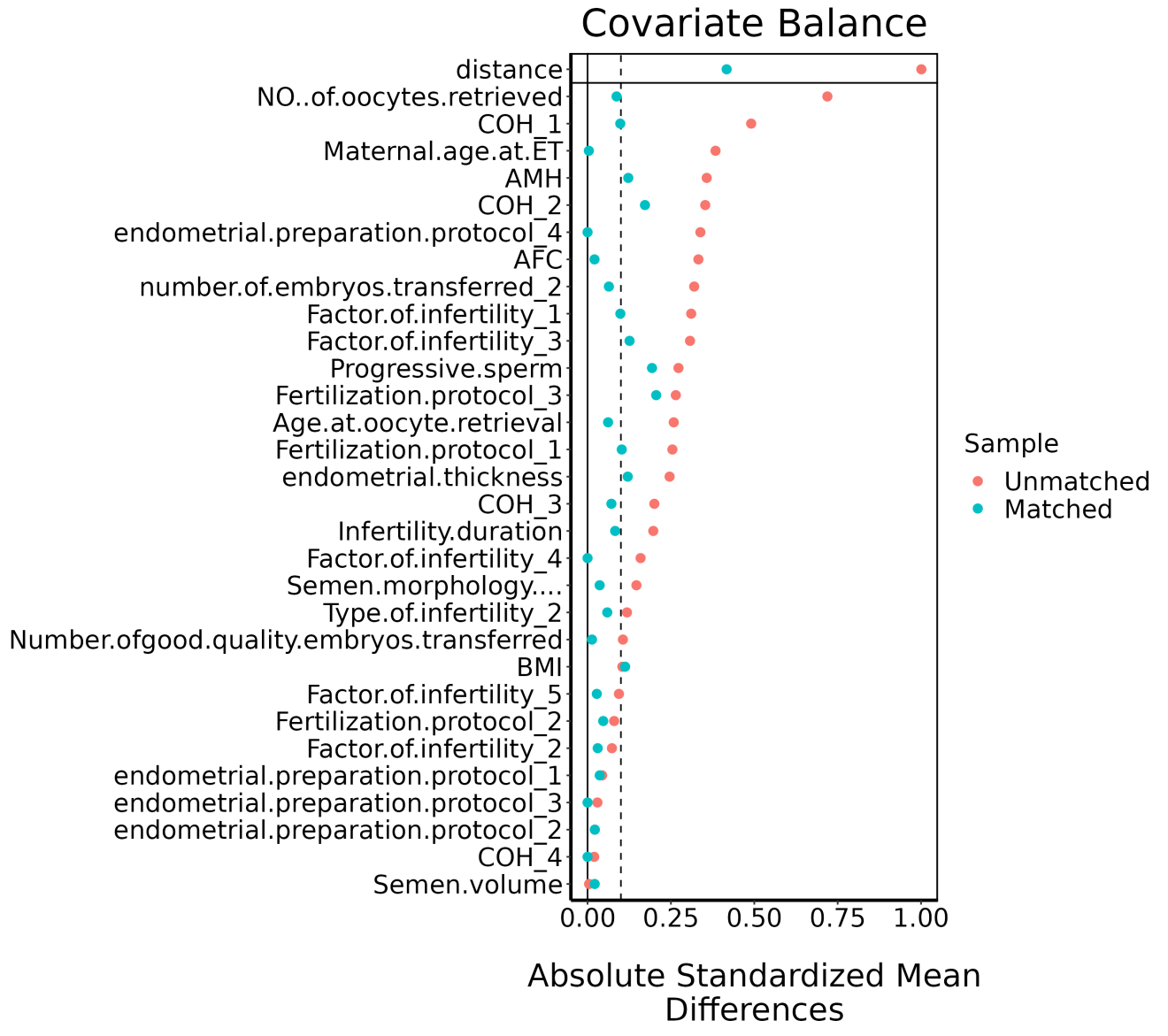


**Supplementary fig 1. Scatterplot of standardized differences for each covariate for patients with an age ≤35**

**Supplementary table 3 General characteristics before and after matching of patients>35 years old**

| Variables | **Before Matching** | | |  | **After Matching** | | |  |
| --- | --- | --- | --- | --- | --- | --- | --- | --- |
| ＞35 year | Long-term culture group  (n=133) | Short-term culture group  (n=287) | SMD^△^ | *P value* | Long-term culture group  (n=133) | Short-term culture group  (n=133) | SMD^△^ | *P value* |
| Age at oocyte retrieval | 39.86 (3.21) | 39.39 (2.91) | 0.147 | *0.13* | 39.86 (3.21) | 39.93 (3.09) | -0.023 | *0.84* |
| Maternal age at ET | 40.38 (3.17) | 39.77 (2.89) | 0.195 | ***0.04*** | 40.38 (3.17) | 40.41 (3.07) | -0.009 | *0.93* |
| Infertility duration | 6.62 (4.68) | 6.03 (4.62) | 0.124 | *0.23* | 6.62 (4.68) | 6.06 (4.62) | 0.119 | *0.33* |
| Type of infertility, n (%) |  |  |  | *0.23* |  |  |  | *0.63* |
| Primary | 25 (18.8) | 69 (24.0) | -0.134 |  | 25 (18.8) | 22 (16.5) | 0.058 |  |
| Secondary | 108 (81.2) | 218 (76.0) | 0.134 |  | 108 (81.2) | 111 (83.5) | -0.058 |  |
| Factor of infertility |  |  |  | *0.00* |  |  |  | *0.92* |
| Tubal factor | 54 (40.6) | 147 (51.2) | -0.216 |  | 54 (40.6) | 60 (45.1) | -0.092 |  |
| PCOS | 3 (2.3) | 6 (2.1) | 0.011 |  | 3 (2.3) | 2 (1.5) | 0.051 |  |
| DOR | 62 (46.6) | 81 (28.2) | 0.369 |  | 62 (46.6) | 60 (45.1) | 0.030 |  |
| Endometriosis | 4 (3.0) | 15 (5.2) | -0.130 |  | 4 (3.0) | 3 (2.3) | 0.044 |  |
| Other | 10 (7.5) | 38 (13.2) | -0.217 |  | 10 (7.5) | 8 (6.0) | 0.057 |  |
| BMI (kg/m^2^) | 22.78 (3.08) | 22.96 (2.75) | -0.058 | *0.54* | 22.78 (3.08) | 23.03 (2.43) | -0.083 | *0.45* |
| AFC (n) | 7.79 (5.74) | 9.31 (5.17) | -0.265 | ***0.00*** | 7.79 (5.74) | 7.59 (4.09) | 0.034 | *0.74* |
| AMH (ng/ml) | 1.41 (1.89) | 1.62 (1.78) | -0.115 | ***0.25*** | 1.41 (1.89) | 1.19 (1.56) | 0.113 | *0.31* |
| COH Protocol |  |  |  | ***0.22*** |  |  |  | *0.70* |
| Long GnRH agonist protocol | 21 (15.8) | 64 (22.3) | -0.179 |  | 21 (15.8) | 15 (11.3) | 0.124 |  |
| Antagonist protocols | 34 (25.6) | 85 (29.6) | -0.093 |  | 34 (25.6) | 34 (25.6) | 0.000 |  |
| PPOS | 72 (54.1) | 127 (44.3) | 0.198 |  | 72 (54.1) | 79 (59.4) | -0.106 |  |
| Other | 6 (4.5) | 11 (3.8) | 0.033 |  | 6 (4.5) | 5 (3.8) | 0.036 |  |
| NO. of oocytes retrieved | 5.94 (5.10) | 7.25 (5.02) | -0.257 | *0.01* | 5.94 (5.10) | 5.68 (4.44) | 0.050 |  |
| Semen volume (mL) | 3.10 (1.38) | 3.14 (1.78) | -0.025 | *0.83* | 3.10 (1.38) | 3.13 (1.82) | -0.020 | *0.89* |
| Progressive sperm | 35.59 (17.86) | 35.12 (18.55) | 0.027 | *0.78* | 35.59 (17.86) | 33.29 (17.20) | 0.129 | *0.28* |
| Semen morphology (%) | 3.58 (2.07) | 3.92 (2.35) | -0.162 | *0.13* | 3.58 (2.07) | 3.64 (2.16) | -0.031 | *0.80* |
| Fertilization protocol, n (%) |  |  |  | *0.15* |  |  |  | *0.69* |
| IVF | 98 (73.7) | 223 (77.7) | -0.091 |  | 98 (73.7) | 101 (75.9) | -0.051 |  |
| ICSI | 31 (23.3) | 62 (21.6) | 0.040 |  | 31 (23.3) | 30 (22.6) | 0.018 |  |
| IVF+ICSI | 4 (3.0) | 2 (0.7) | 0.135 |  | 4 (3.0) | 2 (1.5) | 0.088 |  |

**Supplementary table 4 Clinical outcomes of FETs in two groups before and after matching of patients>35 years old**

| Variables | Before Matching | | |  | After Matching | | |  |
| --- | --- | --- | --- | --- | --- | --- | --- | --- |
| ＞35 year | Long-term culture group  (n=133) | Short-term culture group  (n=287) | SMD^△^ | *P* value | Long-term culture group  (n=133) | Short-term culture group  (n=133) | SMD^△^ | *P* value |
| Endometrial preparation protocol (%) |  |  |  | *0.06* |  |  |  | *0.96* |
| Natural cycles | 18 (13.5) | 17 (5.9) | 0.222 |  | 18 (13.5) | 16 (12.0) | 0.044 |  |
| HRT cycles | 82 (61.7) | 189 (65.9) | -0.086 |  | 82 (61.7) | 86 (64.7) | -0.062 |  |
| GnRH agonist-HRT | 30 (22.6) | 70 (24.4) | -0.044 |  | 30 (22.6) | 28 (21.1) | 0.036 |  |
| Other | 3 (2.3) | 11 (3.8) | -0.106 |  | 3 (2.3) | 3 (2.3) | 0.000 |  |
| Endometrium thickness(mm) | 9.33 (1.78) | 9.23 (1.52) | 0.057 | *0.54* | 9.33 (1.78) | 9.40 (1.47) | -0.035 |  |
| Number of embryos transferred |  |  |  | *0.00* |  |  |  | *0.00* |
| 1(%) | 58(43.61) | 69 (24.04) | 0.418 |  | 58(43.61) | 95(35.71) | 0.216 |  |
| 2(%) | 75 (56.39) | 218(75.96) | -0.418 |  | 75(56.39) | 96(72.18) | 0.216 |  |
| Number of good-quality embryos transferred | 1.20 (0.66) | 1.38 (0.71) | -0.286 | *0.00* | 1.20 (0.66) | 1.20 (0.74) | 0.00 |  |
| Implantation rate (%) | 55/208(26.44) | 129/505(25.54) |  | *0.80* | 55/208(26.44) | 54/223(24.21) |  | 0.59 |
| Clinical pregnancy rate (%) | 32/133(24.06) | 69/287(24.04) |  | *0.99* | 32(24.06) | 29(21.80) |  | 0.66 |
| Miscarriage rate (%) | 10/32(31.25) | 24/69(34.78) |  | *0.72* | 10/32(31.25) | 10/29(34.48) |  | *0.58* |


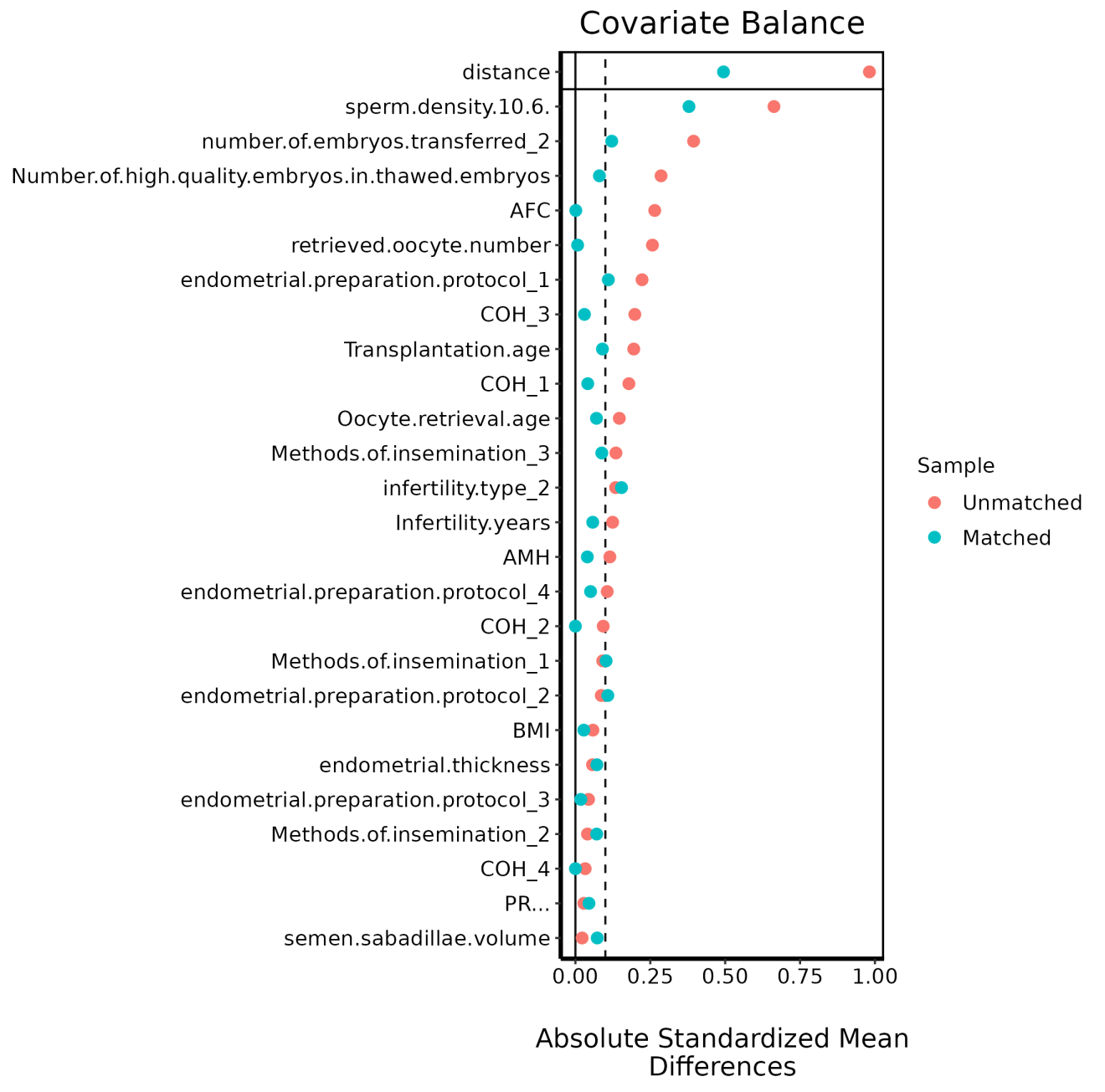


**Supplementary fig 2. Scatterplot of standardized differences for each covariate for patients with an age > 35**
